# Green Auctions and Reduction of Information Rents in Payments for Environmental Services: An Experimental Investigation in Sunan County, Northwestern China

**DOI:** 10.1371/journal.pone.0118978

**Published:** 2015-03-20

**Authors:** Xiaohong Deng, Zhongmin Xu

**Affiliations:** Key Laboratory of Ecohydrology of Inland River Basin, Cold and Arid Regions Environmental and Engineering Research Institute, Chinese Academy of Sciences, Lanzhou, Gansu, China; Chinese Academy of Sciences, CHINA

## Abstract

Reducing information rents is an important task for government agencies wishing to purchase maximal environmental services with limited budgets. This paper reports on several green auction options for reducing information rents and improving the performance of the “Grain for Green” Payments for environmental services (PES) program implemented in northwestern China. In r experimental auctions and investigations, door-to-door interviews were conducted and bidding envelopes and survey questionnaires were used to determine the offers and the foregone profits of the participants. Three scenarios are analyzed in this paper: a uniform price auction, a discriminatory price auction, and an opportunity-cost system. The results show that compared to the uniform price auction system, the other auction systems can increase the cost-effectiveness of conservation contracting. Competitive bidding can reveal true opportunity costs and can reduce information rents extracted from the government by farmers using private information. The demographics and average bids of these auction types were also analyzed. “Perfect information” in the opportunity-cost offer system has the best performance but is very hard to implement in reality. The results of this research show that the auction is a valuable tool for purchasing conservation contracts in northwestern China, but that in the future, the performance of these auctions should be studied with relaxed model assumptions.

## Introduction

With increasing global population, industrialization, agricultural intensification, and economic transformation, serious environmental management issues have arisen, such as water loss and soil erosion, a sharp decrease in global forest areas and subsequent global climate change and increasing land desertification. These environmental and natural resources management issues are an important part of the development challenge in the world, especially in the East Asia and Pacific (EAP) region [1,2]. Payments for environmental services (PES), such as the very large “Grain for Green” program in China, one target of which is Soil and Water Conservation, have become one of the most exciting new environmental policy tools being developed to deal with environmental issues [2,3]. “Grain for Green” is a national ecological project in the central and western regions of China. China’s national “Grain for Green” Project was implemented to protect and improve ecosystems, while enabling an evolution of agricultural management practices compatible with increasing peasants’ incomes. The project attempts to convert agricultural lands on steep slopes or heavily degraded land to forestlands or grasslands.

Without such market-oriented PES systems, faulty information and imperfect markets cause many environmental services to be degraded or lost, even though some people are willing and able to pay for these services [4]. If and only if the ecosystem manager (landowner) has an equal or larger net benefit (i.e., the PES payment is not less than his or her opportunity costs for providing the environmental service), and the beneficiary (e.g., the government) is also ahead because the cost of the PES payment is less than its loss would be if the landowner did not provide the ES, a PES contract between the beneficiary and the landowner can be agreed upon through the market even without government compulsion [1]. Any effective PES system must balance the payment amounts that are possible against the financial requirements of the environmental service (ES) providers.

However, because of information asymmetry between landowners and conservation agents (the beneficiaries) in the markets [5], it is difficult to design and implement a more efficient PES program. If landowners have hidden information or, more characteristically, if information asymmetries exist, adverse selection may result or, alternatively, hidden actions may introduce moral hazard [6]. Therefore, PES payments or compensations must recognize the importance of the specific ES (benefits) indirectly provided by the ecosystem, and more importantly, the opportunity costs of maintaining the service (benefit) should be known.

In PES systems the opportunity costs of providing an ES are the landowners’ private information, which farmers tend to overstate. The difference between the PES payment and the opportunity cost is the information rent. In reality, landowners who are supplying ES have better information than do conservation agents about their opportunity costs, and therefore they can claim higher-than-actual costs in an attempt to extract higher information rents from conservation agents. There are many examples of information rents going to landowners, such as the early U.S. Conservation Reserve Program [7], Germany’s agro-environmental payment programs, and Costa Rica’s Programma de Pagos de Servicios Ambientales (PSA) [8].

Information rents derived from information asymmetry between landowners and conservation agents can limit the effectiveness of PES systems and can make them expensive to implement. Especially if this phenomenon occurs between the government and landowners, information rents will be transferred to taxpayers. In China, the most widely used PES approach relies on available government funds and directs the landowners’ conservation activities, as in China’s “Grain for Green” program which reaches some 30 million farm households and distributes the equivalent of approximately USD $8 billion per year [9]. Therefore, if an effective and efficient PES system is desired (i.e., one in which payments are more than the landowners’ costs and information rents are as low as possible), a policy mechanism must be created to reduce information rents.

Ferraro [5] described three policy mechanisms which can reduce information rents to varying extents in PES programs: (1) gathering more information on landowners in the form of costly-to-fake signals, and then using this information to deduce landowners’ true opportunity costs; (2) screening contracts to differentiate the various types of landowners; and (3) using procurement auctions to introduce competitive forces which reduce information rents. Each mechanism differs in terms of its institutional, informational, and technical complexity and its ability to reduce information rents without distorting the level of ES provided.

One primitive methodology is to acquire information on observable landowner attributes that are correlated with opportunity costs and to use these results to set contract prices. Usually, soil type, distance to main roads, and available markets are correlated with opportunity costs and, importantly, are impossible or costly for landowners to fake. This approach, and sometimes more sophisticated economic models, have been used in U.S. agro-environmental schemes [10,11]. Note, however, that the ability of this approach to reduce information rents is not very good. In theory, screening contracts is a significant improvement that can reveal information in multiple dimensions (e.g., costs, benefits from agricultural activities, and biophysical attributes of ecosystems), including landowner opportunity costs. Designing a menu of contract options that satisfy the participation constraints and the incentive compatibility constraints, and maximize the conservation agent’s desired objectives requires information about landowner type distributions and sophisticated calculations by conservation practitioners. In this mechanism, the conservation agent provides optional contracts, and rational landowners choose one to gain the greats benefit for themselves. Moreover, this action exposes some of the farmers’ information to reduce information rents. This approach is informationally and technically complex to implement [5]. Therefore, although the theory of screening contracts is seemingly perfect, no reported successful cases have been reported in the field to date.

Auctions are a very popular PES method, especially in high- and middle-income nations. Auction theory provides an efficient way to harness competitive forces to reduce information rents. Moreover, auctions have been successfully applied to environmental conservation in agriculture [12–14]. In conservation auctions, a farmer makes a bid that reflects a trade-off between the expected net payoff and the acceptance probability. A higher bid might increase the net payoff, but might reduce the probability of bid acceptance. Therefore, competitive bidding will push farmers to reveal their true opportunity costs and, as a result, will reduce landowners’ information rents, even though the agency acquires limited information about these opportunity costs. However, different pricing rules in auctions (e.g., discriminatory price versus uniform price auctions) can lead to different gains, and auction outcomes are sensitive to the bidding rules and the characteristics of the contracts and the bidders. Because auction theory does not offer clear guidance on appropriate pricing rules, economists have turned to experiments in the field.

This paper uses auctions as a policy mechanism to reducing information rents and examines which pricing rule is most appropriate in the “Grain for Green” auctions of the PES program in northwestern China. To do this, the opportunity costs (the net benefits from their pastures) of landowners in Sunan County, which is in the upper Heihe River in Gansu Province, were investigated. Experimental auctions were conducted to elicit their offers to participate in the “Grain for Green” scheme. The differences among the discriminatory price auction system, the uniform price auction system, and the investigated opportunity-cost system were then analyzed based on the number of participants, the total program outlays, the average bids, and information rents. Because the auctions have not been implemented in the study area, the focus of this paper remains on information rent issues related to hidden information, avoiding moral hazard issues which will be discussed in subsequent work.

## Methods and Materials

**Fig 1:**
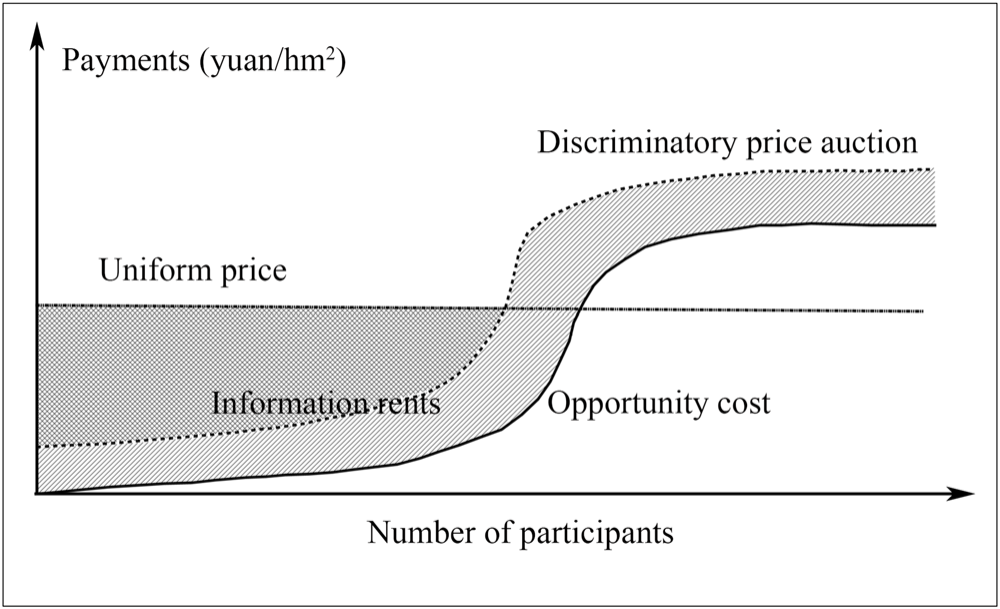
The principle of reducing information rents in the discriminatory price auction system.

A simple example will clarify what is meant by information rent. Assume that a conservation agent would like to contract with a landowner to cease livestock grazing on his pasture to prevent soil erosion. The landowner can obtain value *α*_1_ by continued grazing without the PES contract, but this will lead to environmental loss *l*. In the PES system, assuming the benefit of protecting the pasture is *a*_*0*_ (which often is quite small or even negative), (*a*_1_-*a*_0_) is the opportunity cost of no grazing. From a societal perspective, if *l*>(*a*_1_-*a*_0_) without consideration of the implementation cost, agreeing to the PES contract is efficient. Because (*a*_1_-*a*_0_) is private information of the landowner, the conservation agent cannot know the lowest possible payment to offer in the contract. A rational landowner would report *b* [usually, *b*≥(*a*_1_-*a*_0_)] as the desired payment, as long as there is substantial heterogeneity in the opportunity costs of supplying ES among similar landowners. In this PES system, *r*[*r* = *b*-(*a*_1_-*a*_0_)] is the information rent, which reflects the landowner’s use of his private information as a source of market power to extract a higher payment from the conservation agent. Fig. 1 illustrates why auction systems can reduce information rents. The solid curve at the bottom represents the opportunity cost. Above this line, the dashed curve represents the offer to provide ES, which is the bid in the discriminatory price auction. The above analysis shows that a rational landowner would report a higher price than the opportunity cost. The slash-filled regions between these two lines are information rents. Information rents are difficult to eliminate completely; however, compared to the uniform price system in the “Grain for Green” program in China (the top line), below a certain opportunity cost, the auction bids are lower than the uniform price. Therefore, the slash-filled regions between these two lines are the reduction in information rents from the uniform price system by using the discriminatory price.

### Auctioning conservation contracts

The auction mechanism can be a market institution with an explicit set of rules determining resource or rights allocation, and prices on the basis of bids from market participants [15]. In PES programs, an auction can be used to buy ES from the landowners. Under the same set of basic assumptions for each auction form, four basic auction types (English, Dutch, first-price sealed bid, and second-price sealed bid) yield on average the same revenue to the auctioneer (the Revenue Equivalence Theorem) [16–18]. The basic assumptions are that (1) the bidders are risk-neutral, (2) the bidders have independent private values, (3) there is symmetry among the bidders, (4) the payment is a function of the bids alone, and (5) there are zero costs forbid construction and implementation. These basic assumptions will be used in the analysis below.

A green auction has many attributes, and each combination of auction attributes can induce different bidding behavior. Conservation contract auctions usually offer multiple contracts. For multiple contracts, a conservation agent can choose either a discriminatory first-price sealed bid or a uniform price auction. In the first case, the accepted participants (usually the *n* lowest bidders) are rewarded by receiving the payments stated in their bids. In the uniform price auction, all *n* successful bidders receive the same payment, which may be the average foregone profit or generally the lowest rejected price. In a green auction, the agent can choose the simultaneous or sequential format (Simultaneous auction: Each bidder makes an offer only once. Sequential auction: Each bidder has the opportunity to revise his or her offers.). The buyer may have a maximum reserve price per ES quantity or may have a fixed budget which does not consider the quantity of ES service. The maximum reserve price and the other buyer attributes may be common knowledge or be known only by the buyer.

According to auction theory, in the case of multiple contracts with no budget constraints, the optimal auction design requires in addition the use of a reserve price. In other words, the conservation agent is better off setting a maximum acceptable bid so as to induce farmers to reveal their bids honestly [12,16,17]. Latacz-Lohmann and Van der Hamsvoort [12] developed a model of a discriminatory price, multiple-unit procurement auction in which the conservation agent has a maximum acceptable price per contract. They show that the optimal offer (*b*) to the landowners increases linearly with the landowners’ opportunity costs (*a*_1_-*a*_0_) and the landowners’ expectations about the maximum acceptable price $\left(\overset{-}{\beta }\right)$, or at least $\underset{\_}{\beta }$, where $\overset{-}{\beta }(\underset{\_}{\beta })$ denotes the upper (lower) limit of the bidder’s expectations about the bid cap. The optimal-bid formula of a risk-neutral decision maker is:

b=max{(a1-a0+β-)/2, β_}s.t. b>(a1-a0) (1)

Equation (1) shows that a landowner’s expectation about the maximum acceptable price $\left(\overset{-}{\beta }\right)$ is the key factor influencing his optimal bidding strategy, besides (theoretically) the relatively fixed opportunity costs. Therefore, different levels of landowner certainty about the maximum acceptable price would result in completely different bids.

### Study area

The study area is located in Sunan County in the upper Heihe River basin, Gansu Province. Livestock agriculture is the most important economic sector in Sunan County. Seventy-two percent of the county is grassland (1.7 × 10^6^ hm^2^), of which about 1.4 × 10^6^ hm^2^ can be used as pasture. In recent years, approximately 85% of total grassland has been degraded to various degrees; average pasture yield declined from 921 kg/hm^2^ in 2003 to 752 kg/hm^2^ in 2008. All seven villages in Sunan County were enrolled in the “Grain for Green” program in 2004, and by 2008 the government had invested more than 2 billion yuan in this program, the goal of which is to protect grasslands and promote development of artificial pastures to encourage sustainable development of breeding stock and conserve water and soil. In the early stages of the program, payments for pasture land where grazing was forbidden for a defined length of time during the year were at one uniform level, whereas payments for land that had no grazing during the whole year were at another uniform level. However, the grazing capacity of these grasslands varies spatially and changes from year to year because among these villages there are many grassland types, such as warm steppe, high-cold steppe, and so on. Therefore, this payment scheme might be discriminatory for participants who have different opportunity costs.

### Assumptions and scenarios

Participation in the “Grain for Green” program in Sunan County can involve forbidding grazing for a scheduled amount of time each year or forbidding grazing during the whole year. However, to facilitate the analysis, only one behavior was considered here: forbidding grazing during the whole year. Recall from Equation (1) that foregone profit is one of the main determinants of the optimal bid. Use of this bidding model requires additional assumptions about the farmers’ expectations regarding the maximum acceptable payment level. As mentioned above, the farmers’ expectations were treated as external to the model, and the bidders’ expectations about the maximum acceptable price were assumed to be uniformly distributed in the range from minus *c* to plus *c* of the investigated average opportunity cost; and *c* is varied between 0% and 100%(i.e., the values ranged from complete uncertainty to complete certainty about the maximum acceptable price). Next, it was assumed that each bidder faced the same density and distribution function, which created the benchmark assumption of symmetry among bidders. At the same time, the auctioneers were assumed to meet the other basic assumptions of the benchmark model. Moreover, the various payment systems had the same budget constraint, which was approximately equal to 80% of the overall costs of all the farmers studied, assuming an appropriate participation rate.

To select the optimal auction design for northwestern China, several variants were compared in terms of number of participants, total program outlay, and information rents. These variants were chosen as follows.

Scenario 1: Uniform price auction system (flat-rate offer), which means that the payment *p* is fixed by the program administrator at the presumed average foregone profits of all the participants who have positive opportunity costs. The bids are ranked from lowest to highest and are selected until the budget is exhausted. From the rational farmer’s perspective, participation is worthwhile if *a*_0_ + *p > a*_1_. In this paper, this payment scheme serves as the reference against which the other schemes will be compared, and the payment is equal to the last rejected bid. To date, most conservation programs in China use this payment scheme, but the difference is that regardless of whether their opportunity costs are lower than the maximum acceptable price, the chosen farmers must participate in the program, which entails higher monitoring costs.

Scenario 2: Discriminatory price auction system. In this system, farmers submit sealed bids to the government or to some agency. The government agency accepts bids, starting with the lowest bid, until the budget is exhausted. The winning bidders receive the payments stated in their bids.

Scenario 3: Opportunity-cost offer system. This variant is intended to serve as a “perfect information” case and to calculate information rents for the other two variants. It is assumed that the agency can gain accurate information about each farmer’s true opportunity cost, but as is well known, this will entail high costs for gathering materials and for investigation. In this study, the farmers’ opportunity costs were obtained from the investigation that inquired about the farmers’ total income and costs.

### Experimental auction and investigation implementation

The data used in this paper came from two sources: the two experimental auctions and the investigation. The payment *b* in Scenario 1 and the bid in Scenario 2 were determined by the experimental auctions (S1_Appendix A). Only the opportunity cost in Scenario 3 came from the investigation (S2_Appendix B). An auction has the function of reducing information rents, and it was hoped to implement this feature without having to investigate the opportunity cost beforehand. Note that the farmers who participated in the two auctions were interviewed in the investigation because these variants can be compared to each other; otherwise, gathering so many people together is not easy in vast livestock-raising areas. In other words, the same survey samples were used for all three scenarios.

Ethical approval for this study was obtained from the Academic Board of Cold and Arid Regions Environmental and Engineering Research Institute, CAS. This institutional academic committee is responsible for ethical review. Before detailed investigation of each farmer deeply, the investigators read the survey statement to each farmer to help him or her understand the goal of this anonymous study and to gain permission to write academic papers based on the farmer’s data. Completing the questionnaire was considered as “implied consent.”

Other researchers have used several indicators to represent the costs of participating in PES programs, such as land sales or lease prices as indicators of opportunity cost in these schemes [19–23]). The farmers bid the price of leasing the grassland unit area per year as a substitute for the auction bid of participating in the PES program.

Given the scattered living pattern of herdsmen in northwestern China and the difficulty in gathering them together in one place, experimental auction data were collected in 2011 from door-to-door surveys using sealed-price bid envelopes. A four-person investigation group conducted the survey in Sunan County. Different numbers of randomly chosen peasants were selected in four chosen villages (Table 1). Of the 117 questionnaires distributed, 106 were collected, but two of these could not be used in the analysis because the means of livelihood of these two households had changed—they had sold all their livestock in the previous year. Ages of the informants are between 23 and 76. The numbers of those people aged between 23 and 35, between 36 and 50, and between 51 and 76, are 23, 61 and 22, respectively.

**Table 1:** Questionnaire about opportunity costs and auctions in Sunan County.

Village Name	Number of Questionnaires Distributed	Number of Questionnaires Collected	Average Age of Participants
Dahe	25	20	42
Kangle	67	67	43
Mati	18	14	42
Baiyin	7	5	36
Total	117	106	42

## Results

### Investigation of opportunity costs

**Fig 2:**
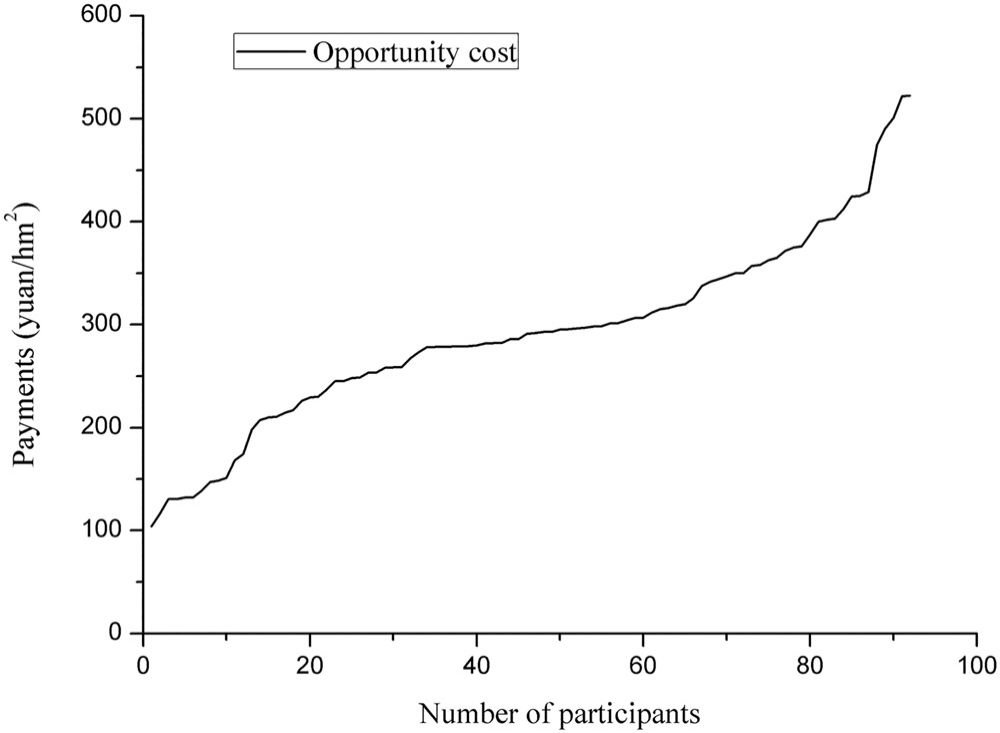
Line chart of opportunity costs for participating in the PES in Sunan County. Near the lower opportunity cost, the slope of this line is less than that near the higher opportunity cost.

As mentioned above, opportunity costs are needed to calculate information rents. Therefore, the obtained opportunity costs were sorted in ascending order and displayed in a line chart (Fig. 2). The minimum value was 103.8 yuan/hm^2^, the maximum value was 774.9 yuan/hm^2^, and the average was 333.7 yuan/hm^2^.

### Characteristics of auction payment systems

The quantitative results of the two experimental auction systems and the opportunity-cost offer system are listed in Table 2.

**Table 2:** Characteristics of the PES program under different payment schemes.

	Uniform price auction system	Discriminatory price auction system	Opportunity-cost offer system
Number of participants	70	83	93
Total program outlays (Ұ)	27580	27387	27376
Average bid (Ұ/hm^2^)	394	330	294
Total information rents (Ұ)	9802	4741	0
information rents (Ұ/hm^2^)	140	57	0

The various payment systems had different performance. Compared to the uniform price auction system, the discriminatory price auction system enhanced performance significantly with almost the same amount of public money. The reasons why the latter auction type was more efficient are the following. First, although the payment mechanism for implementing the uniform price auction system was explained to the bidders, some farmers submitted the same bids as in the discriminatory price auction system. Second, the bidders were encouraged to tender cost-covering bids in these two auction variants, but the payment of the discriminatory price involves less information rent. If opportunity costs only are considered, the latter payment will result in a more efficient system. More information results in better program performance and lower information rents for farmers. As expected, the perfect information (opportunity-cost offer) system had the highest efficiency, more participants, and lower information rents. However, acquiring perfect information is an expensive and risky activity. In this respect, the discriminative-payment auction mechanism can inherently reduce information rents, i.e., benefit losses, more than cost revelation, although the conservation agent has imperfect opportunity-cost values for individual bidders.

It should be mentioned that some participants with lower-than-average opportunity-cost contributions bid less than their opportunity costs as determined in the investigation. This occurred because the lower-than-average bidders’ grasslands consisted of low-fertility lands or high steep slopes. As expected, the quality of grassland affected the quantity of animals that could be carried and therefore the opportunity cost. Moreover, besides the low-opportunity-cost bidders, younger farmers would bid less.

### Difference between the optimal bid and the real auction offer

The theoretical optimal bid is a linear function of the expectation of the maximum acceptable price and the opportunity cost. To obtain the optimal bid, eleven possible maximum acceptable prices were analyzed (Table 3).

**Table 3:** The maximum and the minimum accepted prices under different uncertainties.

Uncertainty	0	10%	20%	30%	40%	50%	60%	70%	80%	90%	100%
Min.	334	300	267	234	200	167	133	100	67	33	0
Max.	334	367	400	434	467	500	534	567	601	634	667

Given the optimal-bid formulas (1), farmers wishing to register low-cost grassland should bid at least $\underset{\_}{\beta }$, whereas high-cost farmers were discouraged from tendering bids and would be rejected from the program. When the farmer knew the reserve price with complete certainty, most of the winners submitted the same bid, equal to $\underset{\_}{\beta }$. Otherwise, a large amount of uncertainty might have induced farmers to bid unreasonably high. According to different degrees of uncertainty about the reserve price, the results of the model calculations are listed in Table 4. All performance figures in the table are assumed to be under the same budget constraint, i.e., if the agent accepted the next higher bid, the total program outlay would be overspent. It is easy to see that when the uncertainty was 10%, the overall performance was better than in the other cases. As uncertainty increased from 10% to 100%, unreasonable bids steadily increased resulting in deteriorating performance, low participation rates, and high information rents.

**Table 4:** The performance of optimal bid according the model under different uncertainties.

Uncertainty	0	10%	20%	30%	40%	50%	60%	70%	80%	90%	100%
Number of participants	82	84	82	79	76	73	70	67	65	63	60
Total program outlays (Ұ)	26625	27583	27559	27654	27710	27685	27573	27374	27559	27691	27759
Average bid (Ұ/hm^2^)	325	328	336	350	365	379	394	409	424	440	463
Total information rents (Ұ)	4393	4535	5327	6612	7790	8850	9796	10629	11478	12247	12947
information rents (Ұ/hm^2^)	54	54	65	84	103	121	140	159	177	194	216

The theoretical model contains a number of basic and added assumptions (see “Assumptions and scenarios”); meanwhile, in the experimental auction, pricing rules for the auction were defined, and only one type of grazing behavior was considered: forbidding grazing during the whole year. In fact, due to the complexity of the real situation and individual differences among landowners, the auction offers and the theoretical optimal bids varied widely. Fortunately, the performance of the uniform price auction system was similar to that of the theoretical strategy under an uncertainty of 60% (in the range of plus/minus 60% of average cost), including the number of participants and the information rents. Moreover, the discriminatory price auction system had an uncertainty close to 10%. Without considering the budget constraint, the average bid difference between the real discriminatory price offer and the model offer under an uncertainty of 40% among those 104 samples was the least. Therefore, the relationship between the difference in the two offers (the discriminatory price auction and the bids under 40% uncertainty) and farmer characteristics was analyzed. The dependent variable was the value of the auction bid minus the model optimal bid for each farmer. The explanatory variables were each farmer’s age, sex, village affiliation (dummy variables) and opportunity cost as determined by the investigation. The model was estimated using simple linear regression. In this situation, a model yielding reasonable predictions was characterized by a coefficient not significantly different from zero and not including the *y*-intercept. The model can be summarized as follows: R^2^ = 0.70, F = 38.72, P = 0.000; and the estimated results listed in Table 5.

**Table 5:** The estimated results of linear regression.

	Standardized Coefficients	t	p
Age	-.405	-1.785	.077
Sex	-.669	-3.653	.000
Opportunity cost	1.921	13.739	.000
Dahe(village1)	-.350	-3.242	.002
Kangle(village2)	-.609	-3.446	.001
Mati(village3)	-.280	-3.137	.002

The results show that the influencing factors analyzed significantly affected (P<0.05) the difference between the real auction bids and the model optimal bids, including opportunity cost, sex, and villages affiliation. Bids from males were less than those from females on average. This may have been because men tended to be risk-averse to increase the probability of acceptance to gain a non-stochastic income component. In China, especially in northwestern China which is poor, the education level of females is very low, and their ability to adopt a new means of livelihood is also low. In this situation, they would be less risk-averse then men, and their offers to participate in the PES program would be higher. As expected, the higher the opportunity cost, the higher was the offer. With respect to village affiliation, farmers from Baiying village offered higher average bids than those from Mati, followed by Dahe and Kangle. The age of bidders was not very significant factor. In fact, the real auction bids were seldom the same as the optimal bids; besides opportunity costs, household and individual characteristics determined the final stated offers.

## Discussion

Without a market to give direct monetary rewards to those individuals who benefit the environment by “doing nothing” and enabling intact ecosystems to continue to provide value, or who change their environmentally destructive behavior or activities, ecosystem managers (landowners) have little economic incentive to improve their environmental stewardship. However, information rents lessen the effectiveness of PES systems. Reducing information rents is an important task for governments that wish to purchase beneficial ES, but have limited budgets.

**Fig 3:**
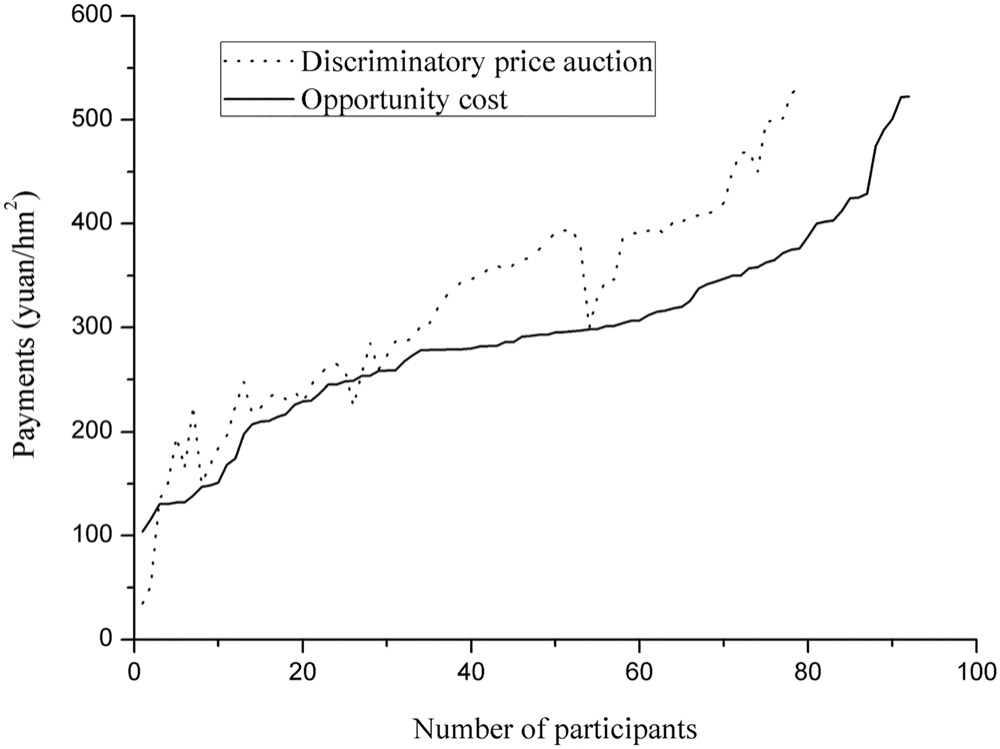
Line chart of the auction bids and opportunity costs for participating in the PES in Sunan County.

This paper has reported on several experimental green auction options for reducing information rents and improving the performance of the “Grain for Green” PES program in northwestern China. It is obvious that in the case of perfect information, the implementation of a bidding scheme would not yield any benefit, and that increasing information would result in better program performance and lower information rents for farmers. However, it is very difficult to determine true opportunity costs accurately, and such investigations are costly, especially when investigating all potential participants in this widespread region. Conversely, auctions that gathered farmers together during the slack season would have lower implementation costs as well as providing benefits from the competition mechanism. As illustrated in Table 5, the bids in discriminatory price auctions were strongly related to opportunity costs, and in this study some of the lowest auction bids were lower than the farmers’ opportunity costs. Moreover, the gap between the auction bids and the opportunity cost expanded from lower to higher bids because the farmers who had lower opportunity costs tended to own poor-quality grassland. Moreover, auction bids did not always increase with opportunity cost (Fig. 3). In some areas, as opportunity costs increased, auction bids became lower. The reasons for this included certain individual characteristics such as sex, age, and the other factors presented in Table 5.

In fact, the changes of livelihoods may influence auction bids of the “Grain for Green” program. Pasture income form only part of the livelihoods of these farmers, many of whom go to nearby towns to perform handicraft work. In other words, they have other sources of income when their land cannot be grazed; some younger people will even work in towns during the whole year. This again leads to deserted villages known as “hollow villages”. Due to rapid urbanization and outmigration of the rural population (especial the younger people), the phenomenon of “hollow villages” has become widespread all over China, including northwestern China [24].It is easy for younger people to change their means of livelihood. They have a higher education compared to older people, therefore new technology acceptability of the former is higher than the latter. These migrated people change their means of livelihood and abandon their grassland or rent it out to left behind middle aged and elderly people. When shifting from traditional pastoralism to a new means of living generates a higher income, a reasonable herdsman would abandon his/her original livelihood and start working elsewhere. So, being forbidden to graze livestock on their grassland in PES program may be a good way to “force” them to find another income source, and subsequently lower their auction bids. The farmers who have new livelihood would bid less and obtain less information rents. In this study, the two participants who submitted bids lower than their opportunity cost were 23 and 24 years old.

As described above, information rents can be seen as returns on private information earned by landowners in addition to the PES payment needed to encourage their participation. The more information the agency acquires, the less farmers will be able to extract high information rents. During auctions, the bidding reveals individual bidders’ opportunity costs of participation to some extent. According to Equation (1), the optimal bid is a linear function of the opportunity cost, and therefore a high bid indicates high opportunity cost and vice versa. Moreover, it is obvious that there is no zero information rent in any auction variant except when the program administrator has perfect information about the foregone profit. However, compared to the uniform price auction system, the discriminatory price auction system has lower information rents. This cost-revelation mechanism and the setting of the maximum acceptable price can reduce “unreasonably” high bids. This makes green auctions a valuable tool for governments to use in PES programs where information asymmetry exists and there are deficiencies in allocating contracts for not providing market goods or services.

The cost of acquiring information may be very high, whether to implement an auction system or to investigate opportunity costs. Therefore, the assumption of the auction model that bid construction and implementation costs are zero may not be accurate. Especially in the “Grain for Green” program in northwestern China, it may be costly to implement a successful auction or an investigation that includes a meaningful number of potential participants. Therefore, the same farmers were chosen to participate in the two kinds of experimental auctions, which may result in a slight bias compared to auctions held separately.

Besides, it is expensive for the farmers to acquire information about the benefits (profits) of participating in the scheme because most of the assumptions in the benchmark model are not grounded in reality. Farmers are generally considered to be risk-averse, not risk-neutral. Accordingly, the conservation payment (as a nonstochastic income component) decreases farmers’ income uncertainty, which induces them to lower their bids marginally to increase the probability of acceptance [12]. It was also found that male farmers are more likely to be risk-averse and therefore to bid less.

Another consideration is the assumption of independent private values, in which each bidder knows precisely how much he or she values the contract in the PES scheme and does not change his or her own bid after learning the valuations of the other bidders. However, grazing income changes every year with the changing prices of market products. In addition, inflation can reduce the value of money provided in the previous year (because the contracts are often signed the year before). Furthermore, the requirement of symmetry among bidders means that all bidders draw their valuations from the same distribution function, but for PES programs, this is not necessarily the case. As mentioned, grassland quality varies by location, resulting in differences in foregone profits as well as potential ES improvements. This study has analyzed the participation constraint and neglected the provision of ES, which means that even if the bids were equal in monetary terms, the resulting provision of ES may have differed. Fortunately, it was found that high bids often correlated with high grassland qualities, which can function better in terms of water and soil conservation. Moreover, and theoretically, in the case of asymmetric bidders, the optimal auction system is generally one in which the item being purchased is assigned to the lowest bidder [16].

Although gaps exist between the model assumptions and reality, the results presented here are meaningful for the government in implementing fair and successful PES programs. To simulate actual auction environments more accurately, in future studies the model assumptions should be relaxed step by step.

## Conclusions

In the PES program, three inefficient phenomena occur due to inappropriate payments provided to participants in the scheme: (1) the payments are higher than the opportunity costs and even more than the value of the ES provided, resulting in uneconomical social costs, high information rents, and eventual negative effects on the efficiency and scale of the whole program; (2) the payments are lower than the losses incurred when farmers change their activities, i.e., the opportunity costs to participate in the program; and (3) inappropriate payments such as a uniform price payment system lead to providing lower compensation for high-opportunity-cost farmers and higher compensation for low-opportunity-cost farmers.

In the “Grain for Green” PES program, some problems in design and implementation exist, such as shortfalls in subsidies delivered, lack of respect of the principals of voluntary participation, and insufficient technical support and budgeting for local implementation costs [25]. One reason for these inefficiencies is that high information rents exist in these programs. Much funding is provided to lower-opportunity-cost farmers, resulting into shortfalls in funding for the program and turning high-opportunity-cost farmers into volunteers. Reducing information rents is an important task for ES buyers, including the government, who have limited budgets, but wish to maximize the services obtained. Government budgets are normally derived from taxes, a fact which implies that inappropriate payments for farmers might induce market distortions. Therefore, any measures that reduce information rents are to be applauded.

This paper shows that auctions are a valuable tool for the government in purchasing conservation contracts from farmers. The various types of auctions have different characteristics; for example, in uniform price auctions, information rents are higher, but implementation costs may be lower, whereas the discriminatory price auction system can reduce some information rents and enroll more participants. Compared to forced participation, auction systems reduce the information rents of lower-opportunity-cost farmers and guarantee fairness among participants. Moreover, auction systems respect the principles of voluntary participation, meaning that the PES program can be implemented over a longer time period and reduce the risk of destruction of grass and trees. It is worth noting that a competitive auction mechanism reduces the possibility of unreasonable bids and of bribes between the local farmers and the officials in charge of the programs. These auction systems can reduce the possibility that farmers subvert the system because the offers are made by the farmers themselves. The various types of auctions provide options for various kinds of PES programs to protect the environment.

## Supporting Information

S1 File Appendix A. Questionnaire used for to determine the price of leasing grassland offered by farmers in Sunan County, northwest China (“auction-like questionnaire”)
(DOC)

S2 File Appendix B. Questionnaire for the opportunity cost in participating payments for environmental services in Sunan County, northwest China
(DOC)
