# Supplementary material for: Green Auctions and Reduction of Information Rents in Payments for Environmental Services: An Experimental Investigation in Sunan County, Northwestern China
Source: PLoS One. 2015 Mar 20;10(3):e0118978. doi: 10.1371/journal.pone.0118978 (PMC4368807; doi:10.1371/journal.pone.0118978)
Supplement: S1 File — Questionnaire used for to determine the price of leasing grassland offered by farmers in Sunan County, northwest China (“auction-like questionnaire”) (DOC) [file pone.0118978.s001.doc]

## Appendix A

**Questionnaire used for to determine the price of leasing grassland offered by farmers in Sunan County, northwest China (“auction-like questionnaire”)**

Dear farmer friend:

We are scientific researcher from Cold and Arid Regions Environmental and Engineering Research Institute, CAS, China. To know your life statement and real thought about PES, we organize this investigation, we hope you can help us.

We are not from the government organization, and our investigation’s goal is to collect your real situation and thought. Your answers and the materials you offered will be used for study and writing academic papers only. Besides, this is an anonymous survey. Please answer all the questions conscientiously. Your answer will be helpful for our research, thank you very much!

Cold and Arid Regions Environmental and Engineering Research Institute, CAS, China

2011.3

(Note: we would told the farmers the payment of the two different auctions and get the double answers.)

Village:_____________

1. You have ____ha grassland in your family.
2. The grassland is used for (optional) __________

① [sheep](app:ds:sheep) [raising](app:ds:raising)____ ② cattle raising____ ③ other animals_____ ④ forbid grazing all year____ ⑤ forbid grazing several months of the year____.

(3) We want to lease your grassland 10/100 ha (according to the real area provided in question (1). How much should we pay you? ____￥.

(4) At this price, how much of the grassland can be leased?____ ha.
