# Supplementary material for: Green Auctions and Reduction of Information Rents in Payments for Environmental Services: An Experimental Investigation in Sunan County, Northwestern China
Source: PLoS One. 2015 Mar 20;10(3):e0118978. doi: 10.1371/journal.pone.0118978 (PMC4368807; doi:10.1371/journal.pone.0118978)
Supplement: S2 File — Questionnaire for the opportunity cost in participating payments for environmental services in Sunan County, northwest China (DOC) [file pone.0118978.s002.doc]

## Appendix B

**Questionnaire for the opportunity cost in participating payments for environmental services in Sunan County, northwest China**

(Note: The statement is same as in the appendix A, and it is omitted here.)

**Part I. Introduction**

1. Brief introduction to the PES program in China (omitted)
2. Questionnaire annotations (omitted)

**Part II. Questionnaire**

We, as member research scientists of the Chinese Academy of Sciences, do not represent any official or governmental organization. We are simply interested in your opinions regarding the opportunity cost in the PES scheme. Your answers will remain anonymous, and you can be assured that your identity will never be revealed. Tick (√) on an option or fill in blanks with your answers, as appropriate. Please answer all questions.

**1. Basic information**

(1) Your sex: 1. Male____ 2. Female____

(2) Your age:____

(3) You have____ ha grassland in your family; ____ha has been used all year; ____ha has been used several months in one year; ____ha has not been used all year.

**2. Income items**

(4) The major source of your income is:

1. Planting____ 2. Grazing____ 3. Working in a nearby town____ 4. Other____

(5) Last year (2010), how many ewes did you have?____; how many did you sell? ____; and how much did each one cost? ____￥.

Last year, how many wethers did you have?____; how many did you sell? ____; and how much did each one cost? ____￥.

Last year, how many lambs did you have?____; how many did you sell? ____; and how much did each one cost? ____￥.

Last year, how many cattle did you have?____; how many did you sell? ____; and how much did one each cost? ____￥.

Last year, how much did you get from selling wool? ____￥

**3. Cost items**

(6) Last year (2010), how much did you pay to buy one [breeding](app:ds:breeding) [sheep](app:ds:sheep)? ____￥; How many did you buy? ____.

Last year (2010), how much did you pay to buy one bull kept for covering? ____￥; How many did you buy? ____.

(7) Operation costs: Forage cost last year ____￥; management fees last year ____￥; treatment fees last year ____￥; deratization fees last year____￥; [shearing](app:ds:shearing) fees last year____￥.

(8) Selling fees: [Transportation](app:ds:transportation) [expenses](app:ds:expenses) last year____￥; slaughter fees last year____￥; casualty losses last year____￥.

(9) Last year, how much land did you use to plant herbage ____ha? Cost to buy seeds ____￥; Cost to buy [chemical](app:ds:chemical) [fertilizer](app:ds:fertilizer)____￥; Cost to buy [pesticides](app:ds:pesticides) ____￥; fees to harvest____￥; fees to transport these herbages ____￥.

(10) Last year, the income of your family was ____￥, of which the income from grazing was____￥, the income from planting was____￥, the income from working in a nearby town was ____￥, the subsidy was____￥, and other income was____￥.
